# P-1398. BCG Vaccine Prevents Primary Tuberculosis in Newborns and School Children

**DOI:** 10.1093/ofid/ofaf695.1585

**Published:** 2026-01-11

**Authors:** Naomi E Aronson, Andrew Hill, Cara Olsen, Joy Abraham, Lee Harrison, Mathuram Santosham, Martin Ottolini

**Affiliations:** Uniformed Services University of the Health Sciences, Bethesda, MD; Uniformed Services University of the Health Sciences, Bethesda, MD; Uniformed Services University, Bethesda, Maryland; 81st Medical Group Keesler Air Force Base, Biloxi, Mississippi; University of Pittsburgh, Pittsburgh, PA; Johns Hopkins, Baltimore, MD; Uniformed Services University of the Health Sciences, Bethesda, MD

## Abstract

**Background:**

Tuberculosis (TB) leads global infectious diseases that cause mortality. Widely used BCG vaccine protects against TB disease (especially miliary, meningeal forms) but the protection of primary TB is inconclusive. Our objective was to assess the effectiveness of BCG vaccine to prevent primary TB using prospectively collected data in a clinical trial.

**Methods:**

This study was approved by the USUHS Institutional Review Board. Data from a controlled trial of BCG (Phipps) in American Indian/Alaskan Native (AI/AN) schoolchildren, n=2763, enrolled 1935-8, and a case control trial of 257 newborns in Rosebud SD, Belcourt ND enrolled 1939-40, were reviewed. Before (schoolchildren only) and after BCG vaccination annual intradermal PPD testing and chest radiographs (CXR) were performed through 1947. Inclusion criteria for this analysis were negative PPD and normal CXR prior to vaccination (schoolchildren), received placebo or BCG vaccine, had a follow-up assessment. Exclusion included reactive or undocumented PPD or abnormal CXR prior to immunization. Primary pulmonary TB was defined as hilar adenopathy, lower lobe infiltrates without other identified etiology, +/- pleural effusion. In the control arm, conversion of PPD >10mm was considered in the context of radiographic findings. Severe TB was miliary or meningeal. Cox proportional hazards regression model with hazard ratios (HR) and 95% confidence intervals (95%CI) was performed for outcomes of primary pulmonary TB and severe TB.

**Results:**

2731 AI/AN schoolchildren, mean age 7.6 years, were evaluated. 1416 received BCG, 1315 placebo. Primary pulmonary TB occurred in 122 BCG, 302 placebo recipients with a sex, age, region-adjusted Cox proportional HR of 0.34, 95% CI 0.28,0.42 for BCG vaccine. 253 newborns were analyzed; 116 received BCG, 137 placebo. For newborns a sex and region adjusted HR for any TB was reported.

**Table:** 

Cohort	Primary TB adjusted HR (BCG)	Severe TB adjusted HR (BCG)
Schoolchildren n=2731	0.34 (95CI 0.28,0.42)	0.24 (95CI 0.08, 0.74)
Newborn n=253	0.36 (95CI 0.13, 0.996)	0 cases BCG, 2 cases placebo

**Conclusion:**

In this heavily TB-exposed AI/AN population, BCG was quite effective in preventing primary pulmonary TB in newborns (64%) or schoolchildren (66%), as well as meningeal or miliary TB (76%).

**Disclosures:**

Naomi E. Aronson, MD, Wolters Kluwer: royalties for writing for uptodate Lee Harrison, MD, GSK: Advisor/Consultant|Merck: Board Member|Pfizer: Advisor/Consultant|Sanofi: Advisor/Consultant

